# Shall Nurses Cease to Be Laywomen?—II

**Published:** 1891-09-19

**Authors:** 


### Passing Topics.

### SHALL NURSES CEASE TO BE LAYWOMEN?—II.

If the recognition of the services of a meritorious body of women were alone to be thought of, the nurses might rely upon the support of every hospital manager for any action intended to advance the interests of the nursing body, and to satisfy their legitimate aspirations.

It is precisely because there is so much to commend in the efforts which have elevated nursing to its present high standard, that it is desirable to examine carefully every new departure which appears likely to disturb the well-understood position nurses occupy in relation to the authorities of the hospital and the doctors. We must not let the notion grow that the hospital exists for the nurses, or that all else is subordinate to their training. Mr. Rathbone * is not alone in his experience, or in the conclusions he draws from it. The over exaltation of the professional element leads people sometimes to forget that the hospitals are maintained by laymen for reasons of pure philanthropy—that is, for the good of the patients, and apart altogether from the training of nurses, or, for that matter, of doctors. The utilisation of our hospitals as schools is legitimate and praiseworthy, but only because the knowledge to be gained there must be supplied somewhere, and is unattainable anywhere else. In an ideal hospital there would be no room for apprentices and learners; we should look for proficiency everywhere.

From the hospital point of view, the flooding of the wards with raw probationers engaged for a limited period, and then set at liberty, is a costly error; it is even more an error when the probationers pay for their board, for this implies a still more limited engagement, both in regard to time and discipline. When we regard the welfare of the patients as our chief care, probationers are but weak vessels, and their numbers relatively to the efficient nurses ought to be strictly limited. Paying probationers should be supernumerary altogether. From the hospital point of view, what is wanted is long service, with a strict sense of discipline. If the aim and object of the novice be not so much to enter upon, and follow the occupation of nursing as to obtain a certificate—that is, if she comes to the hospital in order to qualify for the membership of a "legally-constituted profession" rather than to take a part in its work—then it seems obvious that among other disadvantages, the hospital must look for a shortened period of service on the part of nurses, and consequently for a serious lowering of the standard of discipline and efficiency.

At the outset, the British Nurses' Association proposed to accept as members none but nurses who had completed three years' hospital work, and, so far as it goes, that was a wise restriction. It has been alleged that the rule has not been strictly observed, and that partly-trained nurses have been admitted to membership. If this be so, and an authoritative statement is very desirable, the door is open to the whole body of nurses serving in hospitals. Of course, nurses have the same right as other people to co-operate in any reasonable way to promote their interests, but it is the duty of the hospitals to secure themselves against being made use of by aspirants who either intend to leave the institution directly they become useful and efficient, or, if they remain, will remain less as servants than as members of a profession whose rules, prepared in the interests of the members, may easily conflict with the laws and customs of the hospital.

Agricola.

*(To be continued.)*

* Evidence before the Lords' Select Committee.

